# Differences in self-perception and social gender status in children with gender incongruence

**DOI:** 10.1177/13591045221099394

**Published:** 2022-05-09

**Authors:** Lindsey R van der Vaart, Anouk Verveen, Henny MW Bos, Floor B van Rooij, Thomas D Steensma

**Affiliations:** 1 Research Institute of Child Development and Education, Faculty of Social and Behavioral Sciences, 1234 University of Amsterdam, Amsterdam, The Netherlands; 2 Department of Medical Psychology, Centre of Expertise on Gender Dyphoria, 1209 Amsterdam UMC Locatie VUmc, Amsterdam, the Netherlands

**Keywords:** Self-perception, gender incongruent, social transition, prepubertal children

## Abstract

**Background:** Gender incongruent children report lower self-perception compared to the norm population. This study explored differences in self-perception between children living in their gender role assigned at birth and children living in their experienced gender role.

**Method:** The self-perception questionnaire was administered to 312 children referred to the Center of Expertise on Gender Dysphoria ‘Amsterdam UMC’. Social transition status was determined by parental interviews. 2 (social transition) by 2 (sex assigned at birth) ANCOVA’s were conducted.

**Results:** Children living in their assigned gender role reported comparable self-perception to children living in their experienced gender role. Birth assigned girls living in their assigned gender role reported poorer self-perception on ‘athletic competence’, compared to girls living in their experienced gender role. Birth assigned boys living in their assigned gender role reported poorer on ‘scholastic competence’ and ‘behavioral conduct’ compared to boys living in their experienced gender role.

**Conclusions:** Social transition did not show to affect self-perception. Self-perception was poorer for birth assigned boys living in their experienced gender role. For birth assigned girls this was reversed. Future studies should give more insight in the role of social transitions in relation to child development and focus on other aspects related to self-perception.

## Introduction

According to the American Psychological Association guidelines (APA, 2015), *gender identity* is a persons’ deeply-felt sense of being male, female, or an alternative gender that may or may not correspond to one’s sex assigned at birth. Some children may express a desire to be of another gender than assigned and show gender incongruence like preferring to play with different sex peers, and preferring clothes, toys, and games commonly associated with the other sex (Coleman et al., 2012). Based on population studies regarding problem behavior and social-emotional functioning such cross-gender behavior is observed in approximately 3% of the boys and 5% of the girls. A verbalized desire to belong to the ‘other’ sex is observed in a smaller group, approximately 1.4% of the boys and 2% of the girls (e.g., Steensma et al., 2018).

Children who experience gender incongruence in childhood will not necessarily experience these feelings or will desire a medical transition later in their life (e.g., Olson et al., 2019; Ristori & Steensma, 2016). As such, health professionals who are working with gender incongruent children may be confronted with dilemmas in the counseling. For example, should they support the gender incongruent behavior and affirm cross-gender identity or not? This dilemma is currently one of the most debated aspects in the healthcare for pre-pubertal children who experience gender incongruence and has resulted in different approaches in counseling (e.g., Durwood et al., 2017; Ehrensaft et al., 2018). The need to find more general knowledge on gender incongruent youth and how to guide them is becoming more prominent given that the number of children worldwide referred to specialized gender identity clinics and psychiatric institutions are rapidly increasing (e.g., Byne et al., 2012; Ehrensaft et al., 2018; Olson et al., 2019).

Although there are different approaches in counseling children with gender incongruent feelings, the common denominator seems to be the aim to guide these children the best way possible in their (gender) development and to try to reduce the risk of developing social and emotional problems, including a negative self-concept (e.g., Ristori & Steensma, 2016). Studies show that children with gender incongruent feelings have significantly lower self-concept than the general population (e.g., Alberse et al., 2019).

The view on how to best counsel gender incongruent children has evolved over time and is much under debate. Based on the field Drescher (2013) categorized and described three counseling approaches: (1) the reparative therapy approach, (2) the “watchful waiting” approach, and (3) the affirming approach. The reparative therapy approach is considered unethical to the international standards of care established by the World Professional Association for Transgender Health (2012). In this approach cross-gender identification was considered undesirable and gender identity was seen as malleable through social interventions. The goal of this type of therapy was to attempt to align gender identity with the gender assigned at birth and to decrease the likelihood of hormonal or medical treatment, (e.g., Ristori & Steensma, 2016; Turban & Ehrensaft, 2017). This type of counseling is considered illegal in some States of the USA and different countries worldwide.

In the watchful waiting approach, all gender identification outcomes are equally desirable. This approach states to be reticent in actively initiating an early social transition. Arguments for this statement is that not all children continue to experience gender incongruence and/or desire a medical transition, the likelihood of social stigmatization may increase, and the effect on gender development is unclear. Counselling based on watchful waiting aims to protect children from potential social risk factors but still allows the child to explore one’s gender identity development (e.g., Turban & Ehrensaft, 2017).

Also in the affirming approach, all gender identification outcomes are equally desirable (Drescher, 2013). Affirming-based counselling wants to support and affirm a child’s articulated gender identity expecting the likelihood of developing co-existing pathology to decrease (e.g., Drescher, 2013; Ehrensaft et al., 2018). In this vein, they also support an early social transition of a child if the child’s wish is to live in the experienced gender role and not in the gender role assigned at birth.

The approaches mentioned have different views about an early social transition of a child’s gender role. Knowledge in the literature on the effect of an early social transition is however limited. Clinicians have advised caution to stimulate an early social transition because not all children continue to experience gender incongruence in later life and the uncertainty what the effect of a transition may have on the development of the child (e.g., Byne et al., 2012; Ristori & Steensma, 2016). Furthermore, cases have been described of children who went through a social transition early in life in which the gender incongruence decreased in intensity and desired to transition back to their assigned gender role, which showed to be complicated for some (Steensma et al., 2011; Steensma & Cohen-Kettenis, 2011).

Olson and colleagues (2015) showed that socially transitioned children do not differ in cognitive gender consistency from their cisgender peers and siblings. This entails they are not confused about their gender identity and think of themselves as their experienced gender. A social transition is a choice they make based on clear gender preferences. Furthermore, children who socially transitioned did not differ on self-worth and depression compared to cisgender children; however, they showed elevated rates on anxiety (Durwood et al., 2017).

Considering the above we conclude that there is still a lot to learn about the development of children who go through an early social transition and those who do not. Research on the influence of social transition is needed to shed more light on the concerns mentioned above. In this vein, the current study investigates whether there are differences in various aspects of self-perception between gender incongruent children who went through a social transition and those who did not.

## Methods

### Participants

Current study sample consisted of 312 children referred to the Center of Expertise on Gender Dysphoria at the Amsterdam UMC (location VU), the Netherlands, between 1997–2016. Of the 312 children (*M*_age_ = 9.4, *SD* = 1.2), 37.8% (*n* = 118) were living in the gender role of the sex assigned at birth (28 females and 90 males), and 62.2% (*n* = 194) in their experienced gender identity (137 assigned sex at birth were female and 57 male). The parents of all children provided informed consent to use data collected as part of the intake, diagnostic, and counseling for scientific research. Official approval of this study by our medical ethical commission was not required as the Medical Research Involving Human Subjects Act (WMO) does not apply to the study. Informed consent procedure and data handling was done following the Guidelines for Good Clinical Research Practice.

### Measures

#### Background information

##### Demographics

Questions about age, sex assigned at birth and whether the participant lived in a two or single-parent family were included in the application form of the clinic. The Wechsler Intelligence Scale for Children (Wechsler et al., 2005), was standardized administered in one of the first meetings in the clinic. Intelligence was added as a demographic factor because it is related to psychological functioning and could influence self-perception (Flouri et al., 2018).

##### The intensity of experienced gender incongruence

To measure the intensity of experienced gender incongruence the Gender Identity Interview (GII) was used. The original interview instrument was developed and validated by Zucker et al. (1993). For the purpose of this study the validated Dutch version of the GII was used (GII-C; Wallien et al., 2009). This interview was part of the diagnostic trajectory and includes 12 questions. Four questions are related to cognitive gender incongruence (e.g., “When you grow up, will you be a Mommy or a Daddy”) and eight to affective gender incongruence (e.g., “In your mind, do you ever get mixed up and you are not really sure if you are a boy or a girl?”). Cognitive gender incongruence refers to a situation in which a child mislabels their gender or lacks constancy and affective gender incongruence to the desire to be a member of the other sex. Each question is scored on a 3-point scale, ranging from 0 to 2. An answer was coded with a "0" when the answer was in accordance to the child’s sex assigned at birth (e.g., Are you a boy or a girl?”) or when the child gave a stereotypical response (e.g., “no” to the question “In your mind, do you ever think that you would like to be a [opposite sex]?”). A "1" was coded to an answer when the child provided an ambiguous or intermediate response such as “I do not know” to the question “Do you think it is better to be a boy or a girl?”. When the answer of the child was “Sometimes” to, for example, the question “In your mind, do you ever think that you would like to be a [opposite sex]?”) the answer was also coded with a " 1". An answer was coded with a “2” when it was in line with the desired gender and without ambiguity (e.g., “yes” to the question “In your mind, do you ever think that you would like to be a [opposite sex]?”). Cronbach’s alphas were .75 and .79 for the cognitive incongruence and affective incongruence subscale, respectively. The scores on both scales are based on the sum scores of the items belonging to the scale. Higher scores on the scales can be interpreted as more gender incongruent.

##### Social transition

From 1997 till 2012, the parental interviews at intake included questions about their child’s social transition. The interview questions were about the child’s clothing style and affirmed use of pronouns (e.g., he/him, she/her, they/them) and name. In 2012 these interview questions were formalized in a checklist. Children who were using a name and pronouns and wearing clothing and hairstyle congruent to their assigned sex at birth were categorized as children living in their assigned sex at birth. Children who presented themselves using a name and pronouns and, or, wearing clothing and have hairstyle incongruent to their assigned gender at birth were categorized as children living in their experienced gender.

##### Self-perception

Self-perception was measured by the Dutch version of the self-perception profile for children (SPPC; Hater, 1985; Veerman et al., 1997). This questionnaire consisted of 36 items. An item described two statements. For example, “Some children often forget what they learn BUT other kids can remember things easily.” First, a child had to decide which statement is most relevant for the child, and after that, the child had to rate the degree of similarity with their situation or feelings (“sort of true for me” or really true for me.” The answers on the items were scored on a 4-point scale (1 = low perceived competence – 4 = high perceived competence), a higher score indicating a better-perceived self-perception. The items are grouped into six subscales with in each of them six items: (1) scholastic competence (e.g., “Some kids feel that they are very good at their school work BUT other kids are worried about whether they can do the school work assigned to them”), (2) social competence (e.g., “Some kids find it hard to make friends BUT other kids find it is pretty easy to make friends”), (3) athletic competence (e.g., “Some kids do very well at all kinds of sports BUT Other kids do not feel that they are very good when it comes to sports), (4) physical appearance (e.g., “Some kids are happy with the way they look BUT other kids are not so happy with the way the look”), (5) behavioural conduct (e.g., “Some kids often do not like the way they behave BUT Other kids usually like the way they behave”), and (6) global self-worth (e.g., “Some kids are often unhappy with themselves BUT other kids are pretty pleased with themselves”). The scale scores were based on the mean scores, and Cronbach’s alphas ranged between .72 (athletic competence) and .82 (global self-worth). Percentile scores were computed based on the means and were used in the analyses for each subscale.

### Analyses

Not all children in the age range of 7–13 referred to the Center of Expertise on Gender Dysphoria at the Amsterdam UMC (location VU), the Netherlands between 1997–2016 are included in the current study. Because some children (*n* = 166) who visited the Center for a diagnostic trajectory stopped visiting the Center after one or two sessions mainly because they had enough support in their environment to deal with their feelings. The measurement of self-perception took place during a psychological assessment session, generally after 2 or 3 sessions. Data on background information such as demographics, the intensity of experienced gender incongruence, and social transition status were part of the diagnostic trajectory. To investigate the representativeness of the sample, preliminary analyses were first conducted. In these analyses, background information was compared between the current sample and the children who stopped visiting the Center. As part of the preliminary analyses, we compared within our study sample the background information between those who are living in the sex assigned at birth (*n* = 118) versus those who are living in the experienced gender identity (*n* = 194). Regarding these preliminary analyses, a series of analysis of variance (ANOVA) were used to compare age and mean scores between groups and chi-square analyses to compare percentages.

To examine whether there are differences in the various aspects of self-perception between children living in the gender role align with their assigned sex at birth versus those living in the experienced gender role, 2 (social transition) by 2 (sex assigned at birth) analyses of variances were carried out. When background information variables were significantly associated with social transition status, they were included as covariates in these analyses.

## Results

### Preliminary analyses on background information

Table 1 shows the figures regarding comparing the background information between the 312 children who are part of current study sample and the 166 children who stopped visiting the Center or when data was unavailable. It revealed that the group of children who were included in the study sample, consisted of more assigned females at birth, and showed higher scores in the gender identity interview on cognitive and affective gender incongruence, compared to the children who were not included in the study. Furthermore, children included in the study sample were also living in their experienced gender role more often than children who were not included. No further significant differences in age, intelligence, and family type were observed between the two groups.

**Table 1.** Background information of gender incongruence children referred to the Center of Expertise on Gender Dysphoria separately for those who participated in the current study on self-perception versus those who stopped visiting.

			Sample of the current study versus children who did not remain to visit the center	
	Sample of the current study	Children who did not remain to visit the center^a^	*F*/*X*^2^	*p*
**Demographics**				
Age, *M* (*SD*)	9.4 (1.2)	9.3 (1.5)	1.3	.251
Sex assigned at birth, *n* (%)			6.8	.009
Female	165 (52.9)	67 (40.4)		
Male	147 (47.1)	99 (59.6)		
Intelligence, *M* (*SD*)	101.2 (14.3)	99.2(15.5)	0.1	.721
Family type, *n* (%)			1.0	.323
Two parent family^a^	249 (80.8)	120 (76.9)		
Not in two parent family^2^	59 (19.2)	36 (23.1)		
**Intensity of experience gender incongruence,** *M* (*SD*)				
Cognitive gender confusion	2.5 (2.42)	1.6 (2.36)	12.1	.001
Affective gender confusion	10.3 (4.22)	8.8 (4.54)	10.0	.002
**Social transition status, *n* (%)**				
Living in gender role related to sex assigned at birth	118 (36.8)	114 (71.7)	48.4	<.001
Living in experienced gender role	194 (62.2)	45 (28.3)		

^a^No data available on self-perception.

Children living in their experienced gender identity were significantly older and reported higher scores on cognitive and affective gender incongruence (see Table 2) compared to the children living in their assigned gender role. There were no significant differences in intelligence and family type between the two groups.

**Table 2.** Background information and social transition status of gender incongruence children of the sample of the current study.

	Social transition status						Living in gender role as assigned at birth versus lived in experienced gender identity		Sex assigned at birth female: Living in gender role as assigned at birth versus lived in experienced gender identity		Sex assigned at birth male: Living in gender role as assigned at birth versus living in experienced gender identity	
	Children living in the gender role related to the sex assigned at birth			Children living in the gender role related to experienced gender identity								
	Sex assigned at birth: Female	Sex assigned at birth: Male	Total	Sex assigned at birth: Female	Sex assigned at birth: Male	Total	*F*/*X*^2^	*p*	*F*/*X*^2^	*p*	*F*/*X*^2^	*p*
Demographics												
Age, *M* (*SD*)							8.4	.004	0.4	.547	4.0	.047
*M*	9.4	9.1	9.1	9.6	9.5	9.5						
*SD*	1.3	1.2	1.3	1.1	1.2	1.1						
Intelligence, *M* (*SD*)							1.8	.925	1.3	.261	0.2	.645
*M*	104.8	99.9	101.1	101.3	101.0	101.2						
*SD*	16.3	14.4	15.0	14.4	13.1	14.0						
Family type, *n* (%)							0.0	.861	0.0	.992	0.0	.862
Two parent family^1^	23 (82.1)	71 (79.8)	94 (80.3)	111 (82.2)	44 (78.6)	155 (81.2)						
Not in two parent family^2^	5 (17.9)	18 (20.2)	23 (19.7)	24 (17.8)	5 (08.9)	36 (18.8)						
Intensity of experience gender incongruence, *M* (*SD*)												
Cognitive gender confusion							52.7	<.001	7.9	.006	49.1	<.001
*M*	1.7	1.2	1.3	3.1	3.8	3.3						
*SD*	2.3	2.0	2.1	2.3	2.2	2.3						
Affective gender confusion							47.3	<.001	2.9	.092	34.4	<.001
*M*	10.1	7.8	8.4	11.4	12.0	11.6						
*SD*	4.3	4.3	4.4	3.5	3.6	3.5						

When the background information was only investigated for those assigned at birth as females, it revealed that there was a significant effect on cognitive gender incongruence. Those living in their experienced gender identity showed higher scores on this aspect of the gender identity interview than those living in the gender role related to the sex assigned at birth. No significant differences were found on other background variables for females (assigned at birth) who were and were not living in their experienced gender identity (see Table 2).

For those who were assigned at birth as males, it was found that social transition status was significantly related to age, cognitive and affective gender incongruence. Children who were assigned at birth as males and living in the experience gender identity were, compared to those living in the identity assigned at birth, older, and reported significantly higher scores on cognitive and affective gender incongruence. No significant differences were found on other background variables for males (assigned at birth) who were and were not living in their experienced gender identity (see Table 2).

### Social transition status and self-perception

Table 3 shows the findings from the series of 2 (social transition status: 1= living in gender role related to sex assigned at birth, 2 = living in the experienced gender identity) by 2 (sex assigned at birth: 1 = female and 2 = male) ANCOVAs with as dependent variables the different aspects of self-perception, namely scholastic, social, and athletic competence, physical appearance, behavioral conduct, and global self-worth. In these analyses, age and cognitive and affective gender incongruence were entered as controlling variables because preliminary analyses showed that these variables were significantly associated with social transition status.

**Table 3.** Self-perception and social transition status of gender incongruence children of the sample of the current study.

	Social transition status						Main effect: Social transition status		Main effect: Sex assigned at birth		Interaction effect: Social transition	
	Children living in the gender role related to the sex assigned at birth			Children living in the gender role related to experienced gender identity								
	Sex assigned at birth: Female	Sex assigned at birth: Male	Total	Sex assigned at birth: Female	Sex assigned at birth: Male	Total	*F*	*p*	*F*	*p*	*F*	*p*
Scholastic competence							3.9	.061	33.5	< .001^a^	4.00	.047^b^
*M*	59.3	44.1	47.7	60.6	29.5	50.9						
*SD*	29.8	28.1	29.1	32.6	27.3	33.8						
Social competence							0.1	.720	8.8	.003^c^	1.1	.305
*M*	56.4	48.2	50.1	63.5	46.7	58.3						
*SD*	31.8	31.7	31.8	29.5	32.1	31.2						
Athletic competence							.01	.791	32.9	< 001^d^	5.7	.018^e^
*M*	57.9	44.7	47.8	71.0	39.9	61.3						
*SD*	32.1	30.8	31.5	25.8	28.8	30.6						
Physical appearance							0.2	.621	5.7	.018^e^	0.9	.351
*M*	31.2	32.5	32.2	33.8	20.7	29.7						
*SD*	23.9	32.4	30.5	25.8	25.6	26.4						
Behavioral conduct							3.3	.069	2.2	.138	4.5	.035^f^
*M*	40.6	57.3	53.4	43.4	41.9	42.9						
*SD*	29.6	31.3	31.6	31.0	33.0	31.5						
Global self-worth							1.00	.322	0.2	.645	0.0	.954
*M*	29.2	34.7	33.4	29.2	25.0	27.9						
*SD*	29.6	32.4	31.7	30.0	32.8	30.8						

^a^Female: *M* = 38.68, *SD* = 28.58; Male: *M* = 60.38, *SD* = 31.54.

^b^Female: Living in experienced role versus living in gender role assigned at birth: *F* (1, 136) = 0.63, *p* = .802. Male: Living in experienced role versus living in gender role assigned at birth: *F* (1, 135) = 5.21, *p* = .024. Living in experienced gender role: *M* = 29.54, *SD* = 27.25; Living in gender role as assigned at birth: *M* = 55.08, *SD* = 28.12.

^c^Female: *M* = 62.16, *SD* = 29.94; Male: *M* = 47.62, *SD* = 31.74.

^d^Female: *M* = 68.48, *SD* = 27.45; Male: *M* = 47.62, *SD* = 31.74.

^e^Living in gender role as assigned at birth: Female: *M* = 57.89, *SD* = 31.48, Male: *M* = 44.72, *SD* = 30.81, Female versus Male: *F (*1, 110) = 3.29, *p* = .073; Living in experienced gender role: Female: *M* = 70.98, *SD* = 25.76, Male: *M* = 39.94, *SD* = 29.78, Female versus Male: *F (*1, 161) = 48.86, *p* < .001; Female: *M* = 33.33, *SD* = 25.36; Male: *M* = 28.12, *SD* = 30.51.

^f^Female: Living in experienced role versus living in gender role assigned at birth: *F* (1, 136) = 0.44, *p* = .835. Male: Living in experienced role versus living in gender role assigned at birth: *F* (1, 135) = 8.37, *p* = .004.

There was no significant main effect for social transition status on scholastic competence. However, the main effect for sex assigned at birth showed that females reported higher scores on scholastic competence than males. Also, there was a significant interaction effect between social transition and sex assigned at birth. Only for children who were assigned at birth as males, there was a significant effect for social transition status: Those living in the experienced gender identity reported lower scores on scholastic competence than those living in the gender role assigned at birth. This effect of social transition status was not found for children who were assigned at birth as female.

For social competence, there was no significant main effect for social transition status. However, there was a significant main effect of sex assigned at birth: scores from assigned females at birth were significantly higher on social competence than scores from males assigned at birth. There was no significant interaction effect between social transition status and sex assigned at birth.

There was no significant main effect for social transition status on athletic competence. However, sex assigned at birth was significantly related. This was also the case for the interaction between social transition status and sex assigned at birth. Children assigned at birth as females reported higher scores on athletic competence than those assigned at birth as males. However, based on the significant interaction effect, the difference between the sexes assigned at birth were only significant for the children assigned at birth as females and living in their experienced gender identity.

Regarding physical appearance, there was no significant main effect for social transition status. Also, social transition status by sex assigned at birth was not significantly related to physical appearance. Nevertheless, sex assigned at birth was significantly related to physical appearance, with higher scores for females than for males.

For behavioral conduct, there was no significant main effect for social transition status, neither for sex assigned at birth. However, the interaction between social transition status and sex assigned at birth was significant. It revealed that only for those whose sex assigned at birth was male the effect of social transition status was significantly related to behavioral conduct. Children assigned at birth as male living in their experienced gender role showed lower scores on behavioral conduct than those living in the assigned sex at birth.

There was no significant main effect for social transition status on global self-worth. Also, the effect of sex assigned at birth was not significant; neither that of the interaction of social transition status and sex assigned at birth.

## Discussion

Current study aimed to examine if the self-perception between clinically referred gender incongruent children differed between those who did and those who did not go through a social gender transition. No differences were found between children with a different social transition status. Based on previous studies where gender incongruent children showed to be at risk for developing a negative self-concept (Alberse et al., 2019; Balleur-van Rijn, 2013) and other findings where socially transitioned children showed psychological well-being comparable to their peers (Durwoord et al., 2017) one might have expected a difference between children who did and children who did not socially transition. Recent studies, however, show that parental affirmation and family and peer support may be stronger predictors for psychological well-being than going through a social transition (Pariseau et al., 2019; Sievert et al., 2021). Maybe being referred to a specialized gender clinic, like all children in our study, was a reflection of a supportive parental environment. Such an environment may improve self-perception already. As a result, going through a social transition or not might not further improve self-esteem significantly.

Regarding sex assigned at birth some significant findings were found. Gender incongruent children assigned at birth as females generally reported a more positive self-perception than children assigned at birth as males. This is in line with findings by Alberse et al. (2019) and Balleur-van Rijn at al. (2013). MacMullin et al. (2021) found similar findings in their study. They found that gender nonconformity was associated with a higher report of mental health problems among children assigned at birth as males compared to children assigned as females. An explanation for this difference might be that stereotypical masculine traits are more socially valued than feminine traits (e.g., Braun and Davidson, 2017). This could influence the self-perception of gender incongruent children assigned at birth as males.

The stereotypical valuation of masculinity and femininity could also be the explanation why we found interaction effects between social transition and sex assigned at birth. Children assigned at birth as males living in their experienced gender role reported a poorer self-perception on scholastic competence, and behavioural conduct compared to children assigned at birth as males living in their assigned roles. Whereas children assigned at birth as females and living in their experienced gender role scored higher on athletic competence compared to children assigned at birth as females and living in their assigned gender role. Behavioral conduct is an interesting concept to highlight as it is related to perceived normative and moral behaviour. It might be that assigned males at birth who have socially transitioned perceive their transition as socially unacceptable, contrary to the norm, making them feel more disobedient than assigned males at birth who have not socially transitioned. Children assigned at birth as males who have socially transitioned might experience more restrictions on their gender expressions. Oppositional behaviour, anger, and sadness precipitate specifically when restrictions on gender expression and gender identity are attempted (Chen et al., 2017).

### Limitations and suggestions for future research

A general limitation in research on gender identity expression is the lack of validated instruments and the use and interpretation of terminology. The available instruments have substantial limitations (Bloom et al., 2021). Although the GII, the Dutch version of the GIIC for instance, has adequate psychometric properties (Zucker et al., 1993; Walien et al., 2009) and is widely used across all age groups, it has not been revised since it’s been developed. Therefore, the terminology used is non-affirming and outdated (Bloom et al., 2021).

A methodological limitation of our study is that findings are based on a cross-sectional design. In this vein it cannot be ruled out that children who underwent a social transition may have had a poorer psychological well-being beforehand, compared to the children who did not socially transition or visa versa, which makes it difficult to draw causal conclusions about the effect of a social transition on psychological well-being. To avoid this, future studies should use a prospective longitudinal design. In addition, it is advised to include factors that focus on family and peer support. Furthermore, studies with a qualitative design will also be valuable to provide more insight in considerations of parents and children regarding a social transition in childhood related to gender identity and mental health.

### Clinical implications regarding social transition

In this study, the methodological study design, the approach of a social transition was binary. Social transition, however, is not a static choice, nor can it be captured in a singular decision whether to undergo or not. A social transition is a timely and dynamic process (Pullen-Sansfacon et al., 2020) . An individual pathway where children shape and change their gender role and behavior overtime in order to align one’s external appearance more closely to one’s gender identity. Moving away from social expectations navigating one’s gender identity which is highly influenced by contextual factors as well (Austin, 2016). Clinicians should be aware of this in daily practice and need to adjust their counseling accordingly. Our study shows general advice will not be sufficient as we do not know what the effects of different steps of a social transition will have on individual level.

It seems advisable for clinicians to be cautious in formulating and providing general advice regarding a social gender transition. An individual approach on gender identity exploration in clinical practice seems to be best suited and more important to meet the individual needs. This means that in the guidance of gender incongruent youth and their families, clinicians should inform and explain that the exploration of one’s gender identity is a process, a developmental pathway in which all children differ. Many factors, individual, internal, systemic, and external, play important roles for youth to consider steps aligning their birth assigned gender with their experienced gender. Whether this is within or outside the broad spectrum of gender identity or whether this is aligned with their assigned gender at birth or not is a process to find out and may not be a clear pathway for every child. To affirm one’s gender identity exploration is not the same as utilizing an affirmative or watchful waiting approach. Admitting an affirming attitude entails, in our view, creating an open environment where the situation, needs and desires of the child and caregivers are explored and discussed in relation to what we know from studies and clinical experience. And where through the best possible way is sought for the child and family to grow up and develop in a safe and healthy manner.

## Conclusion

To conclude, we did not observe differences in self-perception between clinically referred gender incongruent children who did or did not socially transition. One does need to be aware of the gender differences regarding the effects of social transition. For some children a social transition might be alleviating, where for others it might not. An individualized approach in counseling children and caregivers in the decision to go through (and shape) a social gender transition is in the best interest of the child’s current and future functioning. Much more has yet to be understood about early social transitions in relation to child development in order to derive more suitable general suggestions for practical use.
